# Repair of articular cartilage defects with intra-articular injection of autologous rabbit synovial fluid-derived mesenchymal stem cells

**DOI:** 10.1186/s12967-018-1485-8

**Published:** 2018-05-09

**Authors:** Zhaofeng Jia, Qisong Liu, Yujie Liang, Xingfu Li, Xiao Xu, Kan Ouyang, Jianyi Xiong, Daping Wang, Li Duan

**Affiliations:** 1 Postgraduate Institution, Guangzhou Medical University, Guangzhou, 511436 Guangdong Province China; 2 Shenzhen Key Laboratory of Tissue Engineering, Shenzhen Laboratory of Digital Orthopeadic Engineering, Shenzhen Second Peoples Hospital (The First Hospital Affiliated to Shenzhen University), Shenzhen, 518035 Guangdong Province China; 3 Guangdong Provincial Research Center for Artificial Intelligence and Digital Orthopedic Technology, Shenzhen, 518035 People’s Republic of China; 4 Department of Chemistry, The Chinese University of Hong Kong, Shatin, Hong Kong SAR China; 5 Shenzhen Kangning Hospital, Shenzhen Mental Health Center, Shenzhen, 518035 Guangdong Province People’s Republic of China

**Keywords:** Mesenchymal stem cells, Autologous, Rabbit synovial fluid, Chondrogenic differentiation, Cartilage defect, Cellular therapy, Intra-articular injection

## Abstract

**Background:**

The role of rabbit synovial fluid-derived mesenchymal stem cells (rbSF-MSCs) in cartilage defect repair remains undefined. This work evaluates the in vivo effects of rbSF-MSCs to repair knee articular cartilage defects in a rabbit model.

**Methods:**

Cartilage defects were made in the patellar grooves of New Zealand white rabbits. The rbSF-MSCs were generated from the knee cavity by arthrocentesis. Passage 5 rbSF-MSCs were assayed by flow cytometry. The multipotency of rbSF-MSCs was confirmed after 3 weeks induction in vitro and the autologous rbSF-MSCs and predifferentiated rbSF-MSCs were injected into the synovial cavity. The intra-articular injection was performed once a week for 4 weeks. The animals were euthanized and the articular surfaces were subjected to macroscopic and histological evaluations at 8 and 12 weeks after the first intra-articular injection.

**Results:**

Hyaline-like cartilage was detected in the defects treated with rbSF-MSCs, while fibrocartilage tissue formed in the defects treated with chondrocytes induced from rbSF-MSCs.

**Conclusions:**

Our results suggest that autologous undifferentiated rbSF-MSCs are favorable to articular cartilage regeneration in treating cartilage defects.

**Electronic supplementary material:**

The online version of this article (10.1186/s12967-018-1485-8) contains supplementary material, which is available to authorized users.

## Background

Articular cartilage is a non-vascular and non-innervated tissue. Because of this unusual structure, injured cartilage has poor healing capacity. Therefore, without treatment, most cases of cartilage injuries result in osteoarthritis (OA) [1]. Numerous techniques are commonly used for cartilage repair. Among them, autologous chondrocyte implantation (ACI) has been the gold standard for cartilage repair in the clinic [2]. However, de-differentiation of chondrocytes while culturing cells in vitro compromises efficacy [3]. Thus, alternative cell sources are needed.

Mesenchymal stem cells (MSCs) are promising candidates for cartilage repair because they are multipotent and highly proliferative. MSC based therapies have been widely investigated for cartilage repair in both preclinical and clinical settings [4, 5]. Because there is no risk of immune rejection and host tissue engraftment occurs more readily, autologous bone marrow-derived MSCs (BM-MSCs) are commonly used for cartilage repair. However, conflicting results have been reported for using BM-MSCs in a collagen-induced arthritis mouse model [6].

Synovial fluid-derived MSCs (SF-MSCs) are another well-studied autologous MSC type used for cartilage repair. Compared to BM-MSCs, SF-MSCs can be easily and non-invasively obtained during diagnosis or treatment [7]. The population of SF-MSCs in synovial fluid greatly increases with joint disease and injury [8]. Therefore, SF-MSCs are likely to be an ideal cell source for cartilage repair [9, 10].

Both MSCs and differentiated MSCs can repair cartilage injury after implantation in the lesion. A predifferentiation procedure prior to treatment has had favorable results probably due to similarities to ACI [11]. While the cartilage injury environment may not favor chondrogenic differentiation, MSCs are more active than seed cells in vivo. The paracrine effect of inflammatory factors is more critical for MSC therapy when it comes to inflammatory diseases [12–15]. Junstunlin et al. observed comparable results using MSCs with or without predifferentiation in their animal model, as the predifferentiation procedure may alter the paracrine factors released [16].

In the present study, we injected both autologous rabbit SF-MSCs (rbSF-MSCs) and predifferentiated rbSF-MSCs to the experimental rabbit articular cavity. We first confirmed the therapeutic effects of autologous rbSF-MSCs, and then compared the therapeutic effects of rbSF-MSCs and predifferentiated rbSF-MSCs. We found that predifferentiation weakened the therapeutic effect of the MSCs, which implies that the predifferentiation process alters the paracrine factors released by the cells.

## Methods

### Animals

Eighteen adult New Zealand white rabbits (6 months old and weighing 2 ± 0.5 kg) were used in this study. To minimize distress, the rabbits were housed singly and allowed to move freely in their cages with unrestricted access to water and food. Prior to the animal experiment, rabbits were allowed to acclimate to their cages for at least 7 days.

The animals were randomly assigned to three groups of six. Synovial fluid was obtained from all animals by arthrocentesis and then articular cartilage defects were induced in the femur condyle. After 2 weeks, the rabbits were intra-articularly injected as follows, group 1 (Control group) with saline, group 2 with predifferentiated rbSF-MSCs, and group 3 with rbSF-MSCs. The injections were administrated once a week for 4 weeks.

### Rabbit synovial fluid collection

The hair on an area about 3 × 3 cm in size within the rabbit knee area was shaved using a safety electric shaver. The site was disinfected three times with the povidone iodine solution and 75% ethanol. Sterile drape application was used to thoroughly dry the area. Isotonic saline solution (2 mL) was injected into the rabbit knee joint cavity from the lateral articular space and the knee was moved to full extension and flection several times. Synovial fluid was collected along with the saline solution using a sterile injection syringe. The synovial fluid was labeled corresponding to each rabbit.

### rbSF-MSC isolation and culture

The synovial fluid was filtered through a 40 μm nylon cell strainer (Cell Strainer, BD Falcon) to remove debris within 4 h of collection. The filtered fluid was collected in sterilized centrifuge tubes and then spun at 1500 rpm for 10 min at room temperature. The supernatant was discarded after centrifugation. The pellet was washed with PBS and resuspended with culture medium [DMEM supplemented with 10% of fetal bovine serum (FBS, Sigma, USA), 1% of penicillin and streptomycin (Sigma-Aldrich, USA)] and then plated on 100 mm dishes. The dishes were incubated at 37 °C in a humidified atmosphere containing 5% CO_2_. After 48 h, the non-adherent cells were removed by changing the media. Then the media was changed every 3 days. The cells adhered to the bottom of the flask and cell colonies formed (Passage 1).

After 7–10 days, cells were lifted by 0.05% trypsin (Life Technologies, USA) and seeded to 75 cm^2^ dishes at a density of about 2000 cells/cm^2^. The media was changed every 3 days (P2) until the cells reached 80% confluency. The cells were passaged several times until the amount of cells reached 1 × 10^8^ cells (P5). The cells were used for flow cytometry, multipotency assay, qRT-PCR, and intra-articular injection.

### Immunophenotyping identification of rbSF-MSCs by flow cytometry

The rbSF-MSCs were lifted by trypsin and suspended in PBS containing 5% bovine serum albumin (Sigma-Aldrich, USA) at a concentration of 3 × 10^5^ cells/50 μL. The cells were incubated with mouse anti-rabbit CD44 (Bio-Rad, MCA806GA), CD73 (eBiosciences, 25073180), CD90 (BD Sciences, 554895), CD31 (Antibodies online inc., ABIN153449), CD34 (Gene Tex, GTX28158), and CD45 (Bio-Rad, MCA808GA) monoclonal antibody (mAb) (1:100 dilution) at 4 °C for 1 h. Then the cells were washed with PBS three times and incubated with a FITC-labeled secondary anti-mouse antibody (Alexa Fluor 488-labeled secondary anti-rat antibody for CD34) (1:200 dilution, Invitrogen) at 4 °C for 30 min. The appropriate rabbit isotype antibodies were used as controls. Samples were processed using a FACS Canto II flow cytometer (BD Biosciences, USA) and analyzed with FlowJo software (Tree Star).

### Trilineage differentiation of the rbSF-MSCs

Passage 5 rbSF-MSCs were seeded in a 6-well tissue culture plate with a density of 10^3^ cells/cm^2^. Trilineage differentiation was induced by the MSC osteogenic differentiation medium (MODM, ScienCell, USA), MSC adipogenic differentiation medium (MADM, ScienCell, USA), and MSC chondrogenic differentiation medium without serum (MCDM, ScienCell, USA). For chondrogenic differentiation, we also used the pellet culture of SF-MSCs for the histological staining. 200,000 P5 cells were pelleted by centrifuge at 400*g* to the bottom of the conical tube. Then cell pellets were induced by the MSC chondrogenic differentiation medium. The induction medium was changed every 3 days for 3 weeks. After induction, the cells were further processed for histological and quantitative real-time PCR (qRT-PCR) analysis.

### Histological staining after trilineage differentiation

After osteogenic differentiation, the cells were fixed with 4% formaldehyde for 30 min at room temperature and stained with 1% Alizarin Red (Sigma-Aldrich, USA) for 5 min [17]. After adipogenic differentiation, the cells were fixed with 4% formaldehyde for 30 min at room temperature and stained with Oil Red O (Sigma-Aldrich, USA) for 10 min [18]. After chondrogenic differentiation, the pellet was fixed with 4% formaldehyde for 30 min at room temperature, sectioned (50 nm thickness) and stained with Toduiline blue (Sigma-Aldrich, USA) for 30 min at room temperature [19]. After staining, the cells were washed with PBS and images were captured under a microscope.

### qRT-PCR analysis after trilineage differentiation

Total RNA was extracted by using Trizol (Invitrogen). The RNA was then reverse-transcribed into cDNA by a DNA synthesis kit (TaKaRa, Shiga, Japan). qRT-PCR was carried out using the SYBR Green PCR Kit (TaKaRa, Shiga, Japan). Rabbit-specific primers were used for analyzing the transcription level of *Osteocalcin* and *Runx2* (runt-related transcription factor 2), collagen type II alpha 1 (*Col2A1*) and sex determining region Y-box 9 (*Sox9*), peroxisome proliferators-activated receptor γ (*PPARγ*) and lipoprotein lipase (*LPL*), for the osteogenic, chondrogenic and adipogenic samples, respectively. Glyceraldehyde-3-phosphate dehydrogenase (*GAPDH*) was used as an endogenous reference. The primer sequences used in this study were listed in Table 1. Real-time PCR was performed with a 7500 real-time PCR detection system (ABI, Foster City, CA). The 2^−ΔΔCT^ method was used to analyze the relative gene expression levels using *GAPDH* as an endogenous control.

**Table 1:** Primers used for real-time PCR

Gene name	Primer sequence
GAPDH	Forward: 5′-GGAGAAAGCTGCTAA-3′
	Reverse:5′-ACGACCTGGTCCTCGGTGTA-3′
Runx2	Forward: 5′-TATGAAAAACCAAGTAGCAAGGTTC-3′
	Reverse: 5′-GTAATCTGACTCTGTCCTTGTGGAT-3′
Osteocalcin	Forward: 5′-GTGCAGAGTCCAGCAAAGGT-3′
	Reverse: 5′-CTAGCCAACTCGTCACAGTC-3′
Col2A1	Forward: 5′-CAGGCAGAGGCAGGAAACTAAC-3′
	Reverse: 5′-CAGAGGTGTTTGACACGGAGTAG-3′
Sox9	Forward: 5′-GTACCCGCACCTGCACAAC-3′
	Reverse: 5′-TCCGCCTCCTCCACGAAG-3′
PPARγ	Forward: 5′-GACCACTCCCACTCCTTTGA-3′
	Reverse: 5′-CGACATTCAATTGCCATGAG-3′
LPL	Forward: 5′-TACAGGGCGGCCACAAGTTTT-3′
	Reverse: 5′-ATGGAGAGCAAAGCCCTGCTC-3′

### Establishment of cartilage defects

Rabbits were placed in a dorsal-recumbent position after general anesthesia induced by injecting 3% pentobarbital sodium into the marginal ear vein at a dose of 1 mL/kg. The knee previously used for synovial fluid collection was used for the operation. The hair on the knee area was shaved and the surgical site was disinfected with the povidone iodine solution and 75% ethanol three times. The rabbits’ knee joints were operated on using a medial parapatellar approach. A cylindrical full-thickness cartilage defect (3.5 mm in diameter and 1.5 mm in depth) was created on the trochlear groove using a special drill (Additional file 1: Figure S1A). Then, the joint capsular was sutured and closed layer by layer using absorbable surgical sutures (VICRYL Plus). After surgery, the rabbits were allowed free movement in their cages. The surgical site was disinfected with 0.1% povidone iodine twice a day for 3 days. Wound healing was monitored for 1 week and no infection was observed.

### Injection of rbSF-MSCs and predifferentiated rbSF-MSCs

The rbSF-MSCs were cultured in culture medium or chondrogenic differentiation medium for 3 weeks. Cells were lifted by trypsin and resuspended in saline solution. 500 μL saline solution containing 5 × 10^6^ cells were articularly injected in the knee joint of experimental rabbits using large 18G size needles (BD, USA) at 7, 14, 21, and 28 days after surgery (Additional file 1: Figure S1B). The control group animals were injected with 500 μL saline solution only.

### Macroscopic score and histological evaluation of cartilage repair

After 8 and 12 weeks of intra-articular injection, all rabbits were sacrificed and the operated distal femur condyles were harvested. The specimens were fixed with 10% formalin solution (Sigma-Aldrich, USA) for 24 h and then decalcified for 24 h with a 10% aqueous solution of nitric acid for paraffin embedding (Sigma-Aldrich, USA). All specimens were cut into sections of 4 μm thickness and stained with hematoxylin–eosin,toluidine blue, Col I and Col II (Abcam, UK) for morphological analysis. The gross appearance and histological evaluation of the defect sites were photographed and blindly scored by 3 independent observers based on the International Cartilage Repair Society (ICRS) macroscopic scoring system (Additional file 2: Table S1) and ICRS Visual Histological Assessment Scale (Additional file 3: Table S2) [20–22].

### Statistical analysis

All data are presented as mean ± standard deviation (SD). Statistical analysis was performed using the one-way ANOVA followed by the Turkey’ post hoc test. All experiments were repeated three times. In all groups, *P* values less than 0.05 were considered to indicate a statistically significant difference, and *P* values less than 0.01 and 0.001 were considered highly significant differences. The Graph-Pad Prism version 6.0 was used for statistical analysis.

## Results

### Characterization of rbSF-MSCs

#### Morphology of rbSF-MSCs

**Fig. 1:**
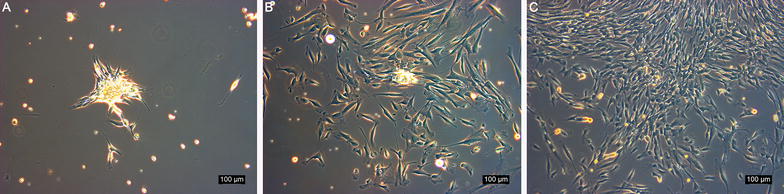
Cell morphology observed by microscope. **A** Colonies formed on the plate (passage 1), **B** Passage 2 cells, **C** Passage 4 cells. Scale bar = 100 μm

Multiple cell colonies formed on the plate after culturing the synovial fluid pellet for several days. The majority of passage 2 cells displayed a spindle-like morphology, but after further passages the percentage of cells with typical fibroblastic cell morphology increased (Fig. 1).

#### Epitope identification of rbSF-MSCs

**Fig. 2:**
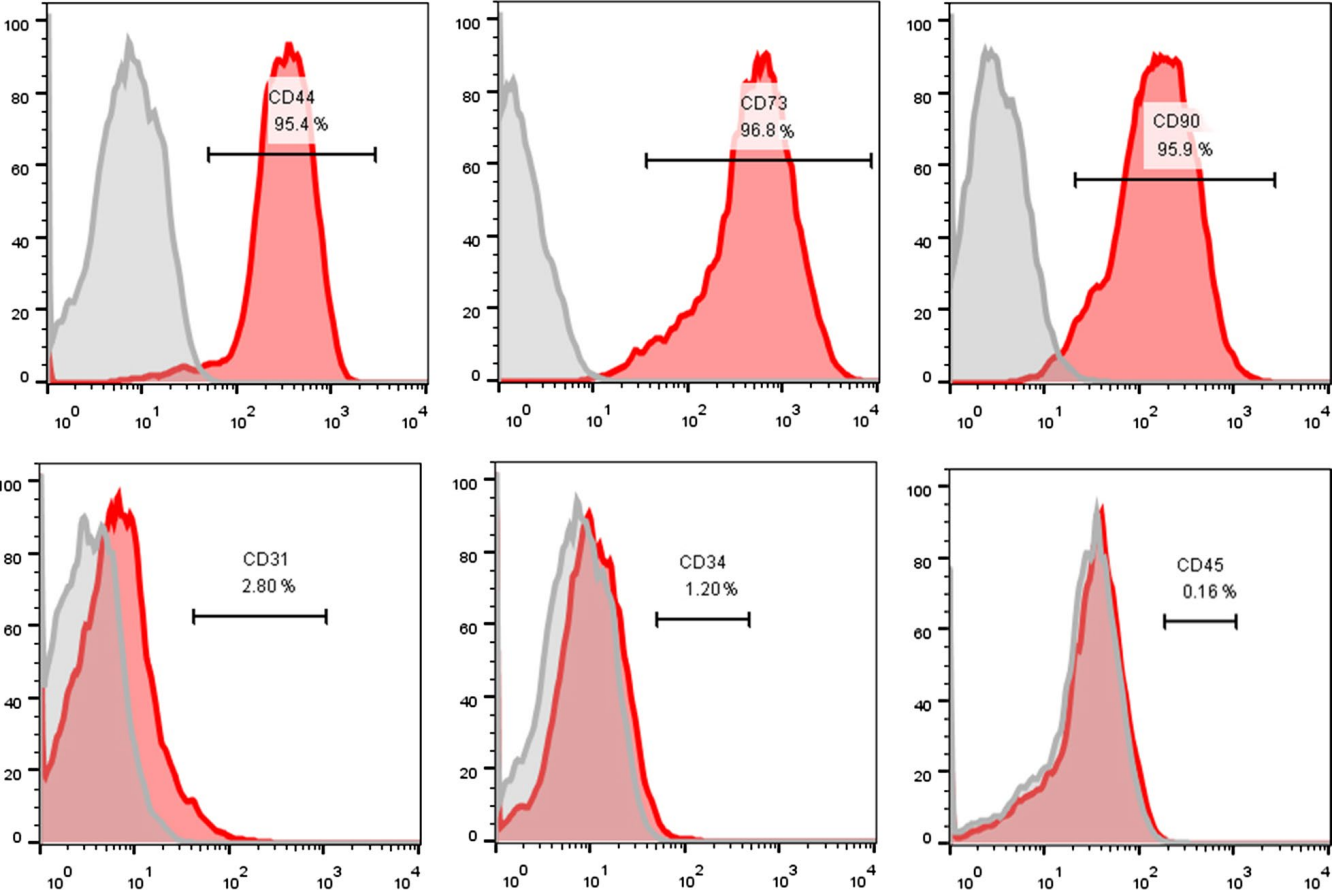
Cell surface marker analysis by flow cytometry. Passage 5 rbSF-MSCs were positive for CD44 (95.4%), CD73 (96.8%), and CD90 (95.4%), and negative for CD31 (2.8%), CD34 (1.2%), and CD45 (0.16%)

Flow cytometry was used to identify the surface markers of rbSF-MSCs, according to the MSC identification criteria recommended by the International Society for Cellular Therapy [23, 24]. The results showed that the rbSF-MSCs we cultured met the identification criteria of MSCs, as the cells were negative for CD31, CD34, CD45 (below 3%) and positive for CD44, CD73 CD90 (above 95%) (Fig. 2).

#### Trilineage differentiation of rbSF-MSCs

**Fig. 3:**
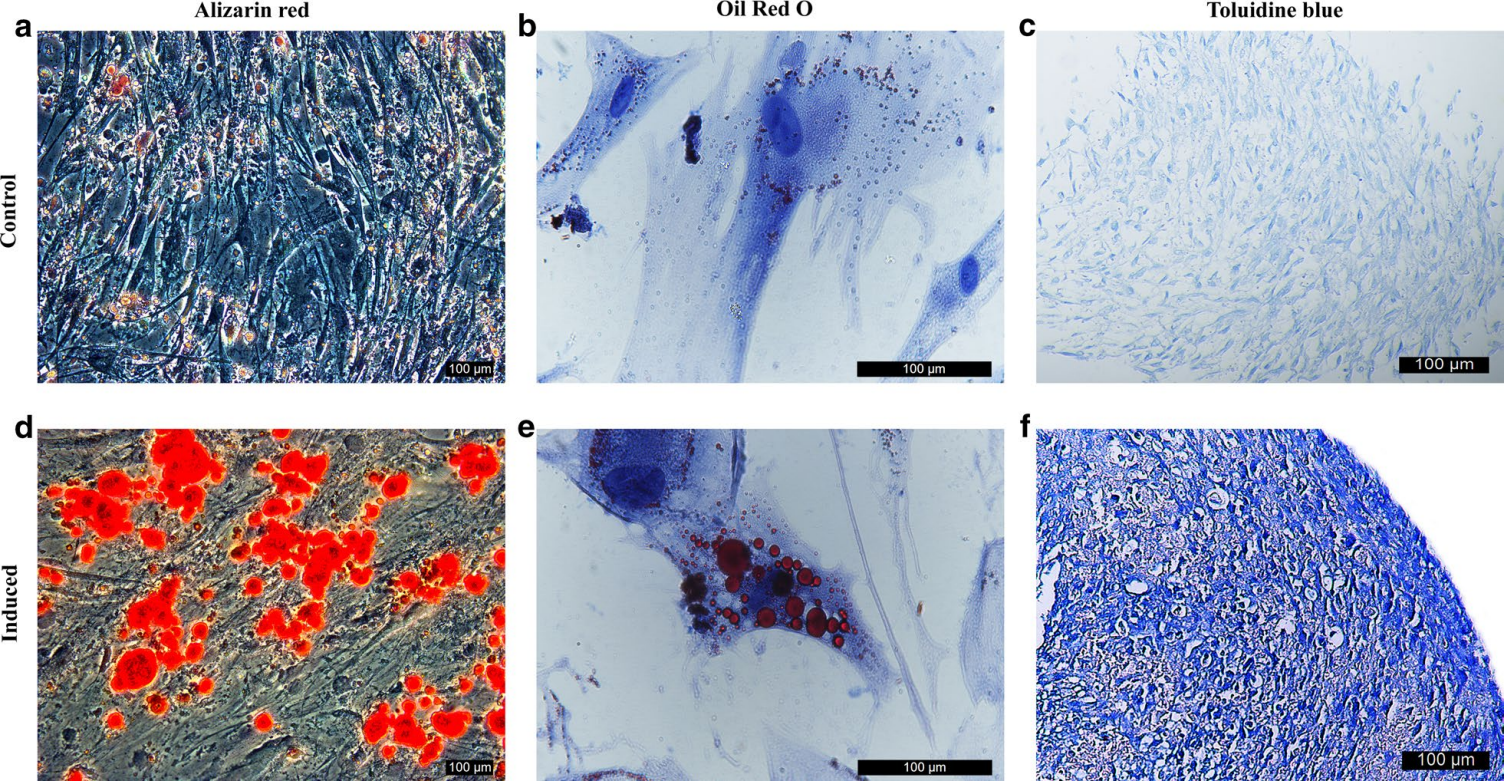
Trilineage differentiation of rbSF-MSCs characterized by histological staining. **a**, **d** Alizarin red staining for osteogenic differentiation; **b**, **e** oil red O staining for adipogenic differentiation; **c**, **f** toduiline staining for chondrogenic differentiation. Scale bar = 100 μm

**Fig. 4:**
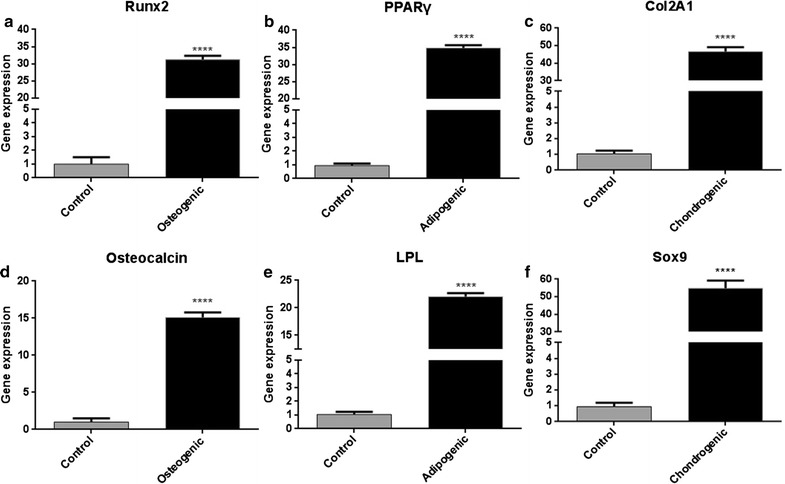
Trilineage differentiation of rbSF-MSCs characterized by qRT-PCR analysis. **a**, **d** Induced rbSF-MSCs had much higher levels of the osteogenic marker gene (Runx2 and Osteocalcin) than control cells. **b**, **e** Induced rbSF-MSCs had up-regulatd PPARγ and LPL compared with control cells. **c**, **f** Chondrogenic differentiation markers (Col2A1, Sox9) were significantly increased after induction compared to the control. Gene expression was normalized to GAPDH, and obtained from at least three independent experiments. (****P < 0.0001)

To further evaluate the stem cell properties of rbSF-MSCs, we assayed their potential to differentiate to osteogenic, adipogenic, and chondrogenic lineages. After culturing the rbSF-MSCs in osteogenic media for 3 weeks, the formation of calcium mineral deposition was clearly observed by Alizarin Red staining (Fig. 3a, d). Also, Osteocalcin and Runx2 were strongly induced when compared to the control cells (Fig. 4a, d). To assess adipogenesis, cells were induced by adipogenic medium for 3 weeks. Oil Red O staining indicated the formation of lipid droplets (Fig. 3b, e). Up-regulation of the adipocyte marker genes, PPARγ and LPL, further confirmed adipogenic differentiation (Fig. 4b, e). When the rbSF-MSCs were cultured in chondrogenic medium for 3 weeks, they successfully differentiated into chondrocytes, which was confirmed by both Toduiline blue staining (Fig. 3c, f) and the up-regulation of Col2A1 and Sox9 (Fig. 4c, f).

### Cartilage repair effects of rbSF-MSCs and predifferentiated rbSF-MSCs

#### Gross observation of repaired cartilage

**Fig. 5:**
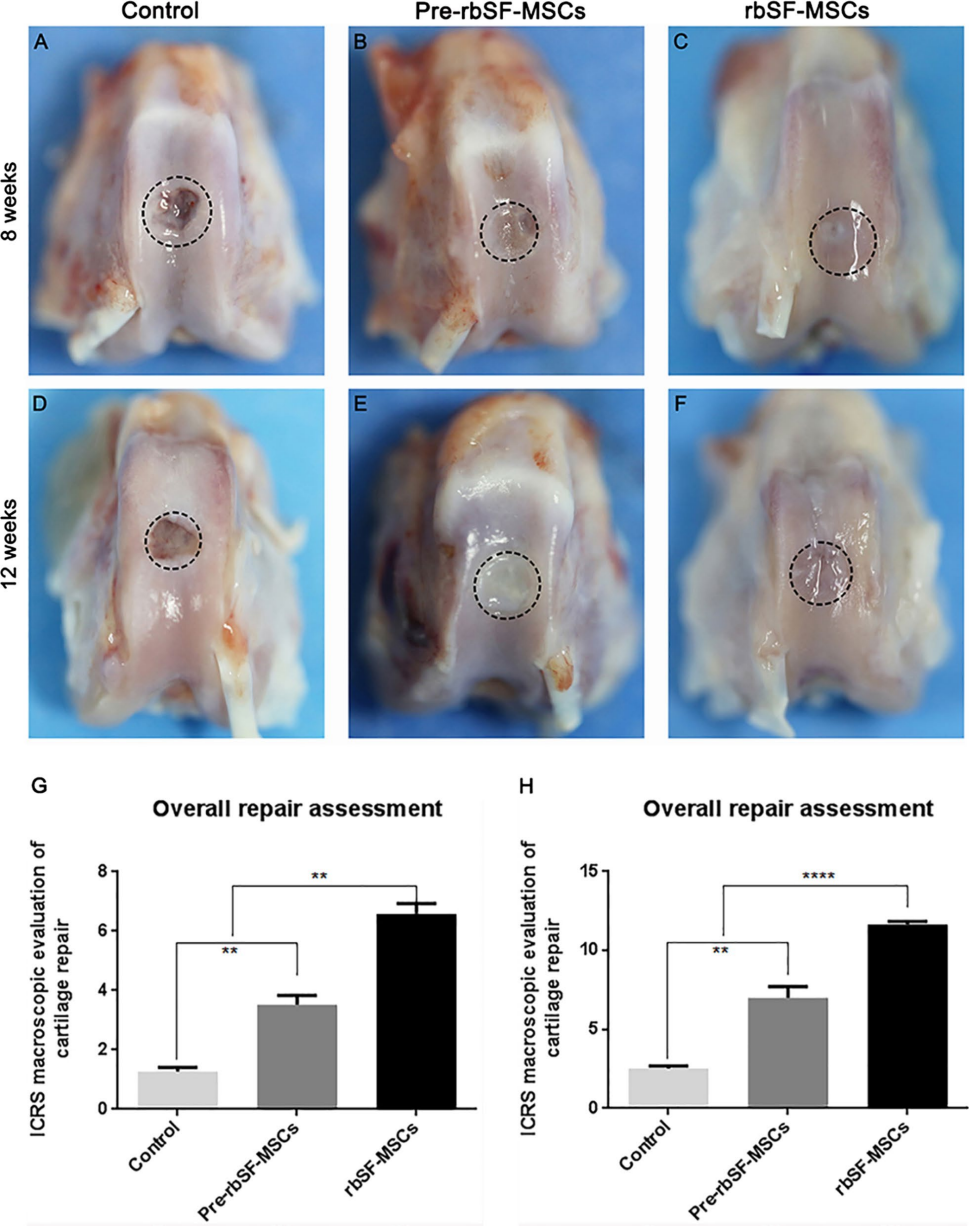
Macroscopic assessment of repaired cartilage. **A**–**F** Photographs of rabbit knee articular defects 8 and 12 weeks after cell injection. Black dotted circles indicate the original defect margin. **G**, **H** ICRS macroscopic scores of repaired cartilage at 8 and 12 weeks. Data are presented as mean ± SD (n = 6, **P < 0.01, ****P < 0.0001)

Rabbits were sacrificed 8 and 12 weeks after the first intra-articular injection and the femoral condyles were harvested. We first evaluated the performance of cartilage repair by gross observation (Fig. 5A–F). The saline-treated control group still had a clear boundary between the normal tissue and the defect after 8 and 12 weeks (Fig. 5A, D). The defects in the predifferentiated rbSF-MSCs group was covered by a thin layer of fibrous tissue at 8 weeks, and was completely covered with white hyper-proliferative fibrous tissue at 12 weeks (Fig. 5B, E). The regenerated tissue in the rbSF-MSCs group covered more than 80% of the defects at 8 weeks and the defect was completely repaired at 12 weeks (Fig. 5C, F). Therefore, rbSF-MSCs exhibited better repair effects than the predifferentiated rbSF-MSCs. We further evaluated repair using the ICRS macroscopic scores. Both the predifferentiated rbSF-MSCs and rbSF-MSCs treatments had much higher scores than the control group at the two time points, indicating that the treatments with both cells were effective (Fig. 5G, H). However, surprisingly the predifferentiated rbSF-MSCs group had significantly lower scores than the rbSF-MSCs group. Therefore, based on gross observation, we found that injection of both cells aids in cartilage repair, but rbSF-MSCs produced better outcomes.

#### Histological analyses of repaired cartilage

**Fig. 6:**
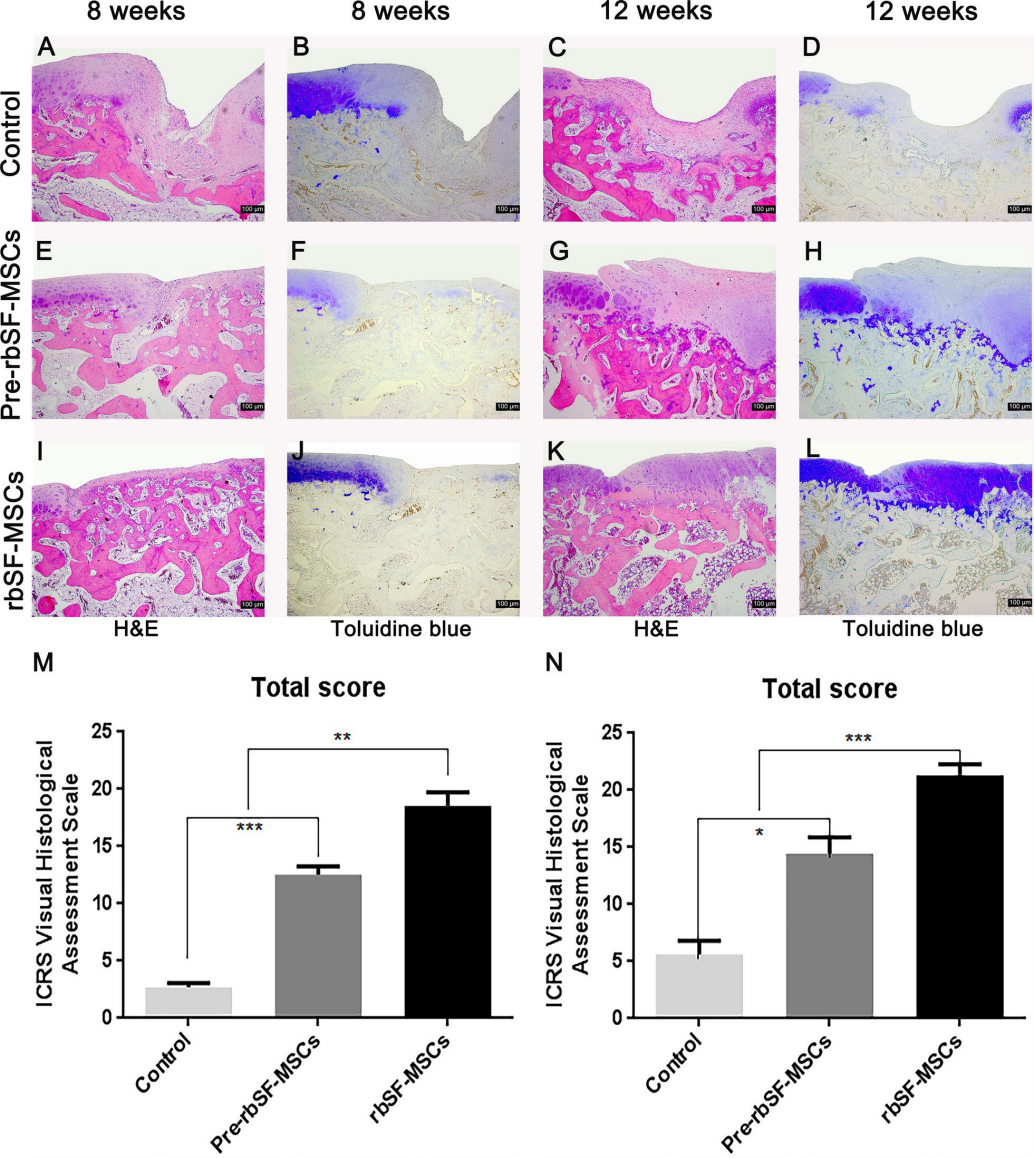
Histological evaluation of repaired cartilage. **A**–**L** Representative H&E and toluidine blue staining of repaired cartilage at 8 and 12 weeks. Scale bar: 100 μm. **M**, **N** ICRS Visual Histological Assessment Scale for repaired cartilage at 8 (M) and 12 (N) weeks. Data are presented as mean ± SD (n = 6; *P < 0.05, **P < 0.01, ***P < 0.001)

**Fig. 7:**
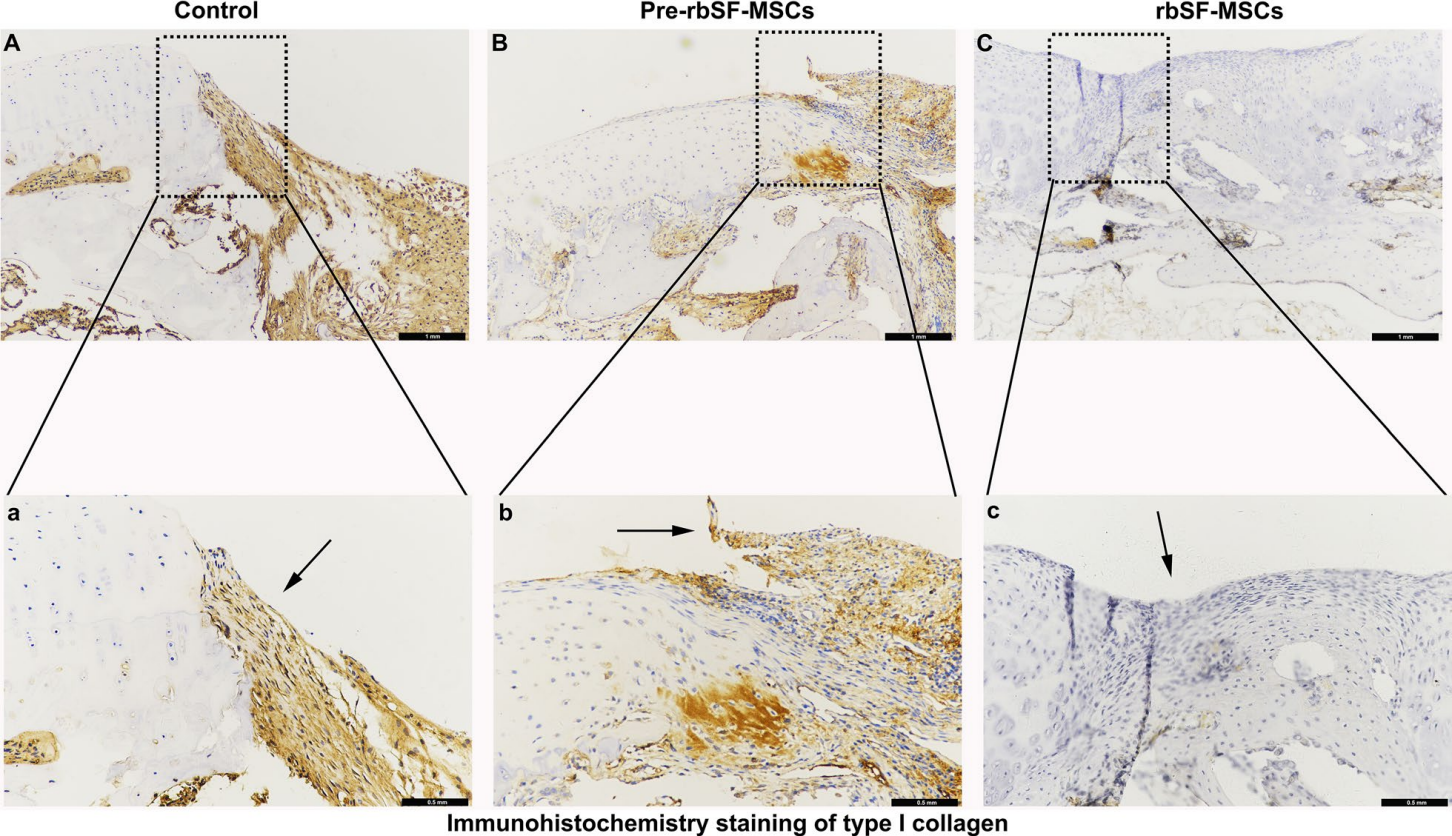
Immuno-histological staining of Collagen I. **A**–**C** Representative Collagen I staining of repaired cartilage at 12 weeks. **a**–**c** Enlarged image of the dashed box in **A**–**C**, respectively. Scale bar: 100 μm

**Fig. 8:**
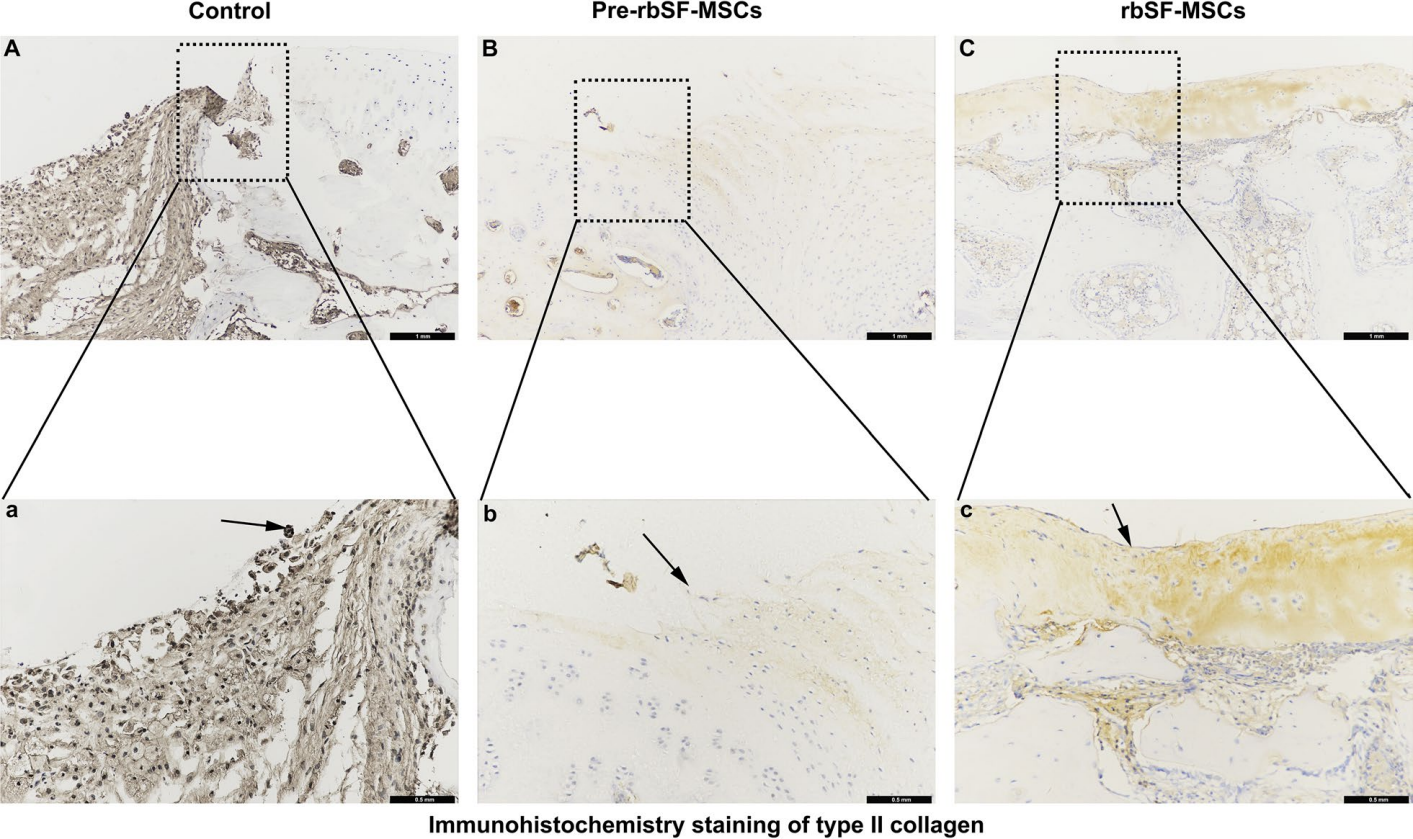
Immuno-histological staining of Collagen II. **A**–**C** Representative Collagen II staining of repaired cartilage at 12 weeks. **a**–**c** Enlarged image of the dashed box in **A**–**C**, respectively. Scale bar: 100 μm

Cartilage damage without intervention resulted in obvious hollowing and the formation of fibrous tissues after 8 weeks (Fig. 6A, B). The damaged area decreased after 12 weeks, and was covered by some inflammatory tissue (Fig. 6C, D). Animals injected with predifferentiated rbSF-MSCs obtained better repair compared with the control group. However, the regenerated tissue was fibrous and the gap between newly grown tissue and native cartilage was obvious at 8 weeks (Fig. 6E, F). After 12 weeks, the regenerated tissue was primarily hyper-proliferated fibrous tissue, while only a small part was cartilage-like tissue as indicated by Collagen I immune-histological staining and Toluidine blue staining (Figs. 6G, H, 7). The rbSF-MSCs group regenerated cartilage better than the predifferentiated rbSF-MSCs group, which was similar to the gross observation (Fig. 6I, J). With the injection of rbSF-MSCs, there was newly-formed cartilage tissue in the defect at 8 weeks (Fig. 6I, J). After 12 weeks, the regenerated tissue completely filled the defect, which had a similar histological staining as the native hyaline cartilage as indicated by Toluidine blue and Collagen II staining (Figs. 6K, L, 8).

We further evaluated the repair effect based on the ICRS Visual Histological Assessment Scale system (Fig. 6M, N). Similar with our other observations, treatment with both cell types resulted in obvious repair compared to the control group. However the rbSF-MSCs showed a significantly higher repair score than the predifferentiated rbSF-MSCs. Overall, the rbSF-MSCs showed the best effect in regenerating cartilage after damage.

## Discussion

Articular cartilage tissue repair is challenging because of the limited self-regenerative potential of native cartilage [25, 26]. MSC-based therapy holds great promise for restoring cartilage defects [27]. SF-MSCs are easily obtained and are highly proliferative, making them an ideal cell source for cartilage repair [28, 29]. In this study, we injected autologous rbSF-MSCs into the experimental rabbit articular cavity. Results of this study showed that the cartilage defect was fully repaired with undifferentiated rbSF-MSCs after 12 weeks. This is the first study to demonstrate the superiority of autologous rbSF-MSC injection for cartilage repair in a rabbit model.

It has been reported that predifferentiation benefits repair because it mimicks ACI [11]. We therefore injected predifferentiated rbSF-MSCs for cartilage defect repair. However, our results showed that the regenerated tissue after injecting predifferentiated rbSF-MSCs was mainly composed of fibrous tissue, which is unfavorable for cartilage repair. Therefore, predifferentiation might hinder the regenerative effects of rbSF-MSCs, which contradicts result reported by others. Several studies have reported that MSCs function in vivo as seed cells and in the production of bioactive factors [30–32]. The predifferentiation process could also weaken the stemness of rbSF-MSCs as well as altering the autocrine and paracrine factors [33–35]. Recently, it’s been shown that exosome released from MSCs can be used to treat inflammatory diseases, including osteoarthritis [36, 37]. Besides osteoarthritis, Zhu et al. reported that a stem cell-derived exosome-laden hydrogel could regenerate cartilage tissue in a rabbit model after 12 weeks, which confirmed the repair effects of the paracrine factors [38].

Carrying bioactive factors may cause the differences between our study and others. Researchers often seed the pre-differentiated cells on a scaffold [39–41]. When implanting the cell-scaffold complex in a defect, they implanted the paracrine factors from the cells and the extracellular matrix together. However, in this study, we digested the cells from the culture flask before injection. Therefore, only the differentiated cells was injected without the paracrine factors. Junstunlin et al. injected resuspended cells for cartilage repair as well, but they administrated cells together with platelet-rich plasma, which had plentiful bioactive factors [16]. They observed comparable repair effects for both undifferentiated MSCs and predifferentiated MSCs. Without the nutrition of the bioactive factors from the platelet-rich plasma, the predifferentiated MSCs may have less repair activity. We remain in need of further investigations to clarify the physiological mechanisms.

Thirdly, the inherent advantages of the SF-MSCs could establish their potential role in cartilage damage treatment. Jones et al. reported that the number of SF-MSCs in knee joints significantly increased sevenfold during the early stages of osteoarthritis (OA) [8]. The increased SF-MSCs could contribute to maintaining the physiological homeostasis of joints. In addition, treatment with autologous SF-MSCs has no dispute about safety and immunogenicity. Furthermore, the SF-MSCs possess highly immunosuppressive properties in vivo [7]. Compared to the BM-MSCs and synovium-derived mesenchymal stem cells (SM-MSCs), SF-MSCs prefer differentiation into functional chondrocytes and secrete a large amount of extracellular matrix, making them an excellent alternative cell source for cartilage regenerative therapy [42–44].

This study also had several limitations. The optimum cell number of rbSF-MSCs for intra-articular injection remains unknown. It is also uncertain whether the newly regenerated tissue was completely induced from injected rbSF-MSCs. Finally, in our animal study, we observed and evaluated results after 8 and 12 weeks. This follow up time may be too short to show more significant differences of the repair quality.

## Conclusions

In summary, we confirmed the repair effects of autologous rbSF-MSCs in a rabbit model. We found that a predifferentiation process was not helpful for cartilage repair, which could be explained by loss of paracrine function of MSCs by predifferentiation. Intra-articular injection of autologous undifferentiated rbSF-MSC represents a better approach for efficient and effective treatment for articular cartilage lesions. This in vivo research could contribute to the use of SF-MSC-based therapeutics in cartilage tissue engineering.

## Additional files

**Additional file 1: Figure S1.** Surgical procedures of rabbit articular cartilage defect introduction and intra-articular injections of rbSF-MSCs. (A) A cylindrical full-thickness cartilage defect (3.5 mm in diameter and 1.5 mm in depth) was created on the trochlear groove using a special drill. (B) The cells were articularly injected in the knee joint of experimental rabbits using using large 18G size needles.

**Additional file 2: Table S1.** International Cartilage Repair Society macroscopic evaluation of cartilage repair.

**Additional file 3: Table S2.** The ICRS Visual Histological Assessment Scale.

## Abbreviations

ACI: autologous chondrocyte implantation
MSC: mesenchymal stem cell
BM-MSCs: bone marrow-derived mesenchymal stem cells
rbSF-MSC: rabbit synovial fluid-derived mesenchymal stem cell
SM-MSCs: synovium-derived mesenchymal stem cells
OA: osteoarthritis
PBS: phosphate-buffered saline
DMEM: Dulbecco’s modified eagle medium
MODM: MSC osteogenic differentiation medium
MADM: MSC adipogenic differentiation medium
MCDM: MSC chondrogenic differentiation medium
FBS: fetal bovine serum
Runx2: runt-related transcription factor 2
Col2A1: collagen type II alpha 1
Sox9: sex determining region Y-box 9
PPARγ: peroxisome proliferators-activated receptor γ
LPL: lipoprotein lipase
GAPDH: glyceraldehyde-3-phosphate dehydrogenase
