# Supplementary figures and images for: Repair of articular cartilage defects with intra-articular injection of autologous rabbit synovial fluid-derived mesenchymal stem cells
Source: J Transl Med. 2018 May 9;16:123. doi: 10.1186/s12967-018-1485-8 (PMC5941664; doi:10.1186/s12967-018-1485-8)

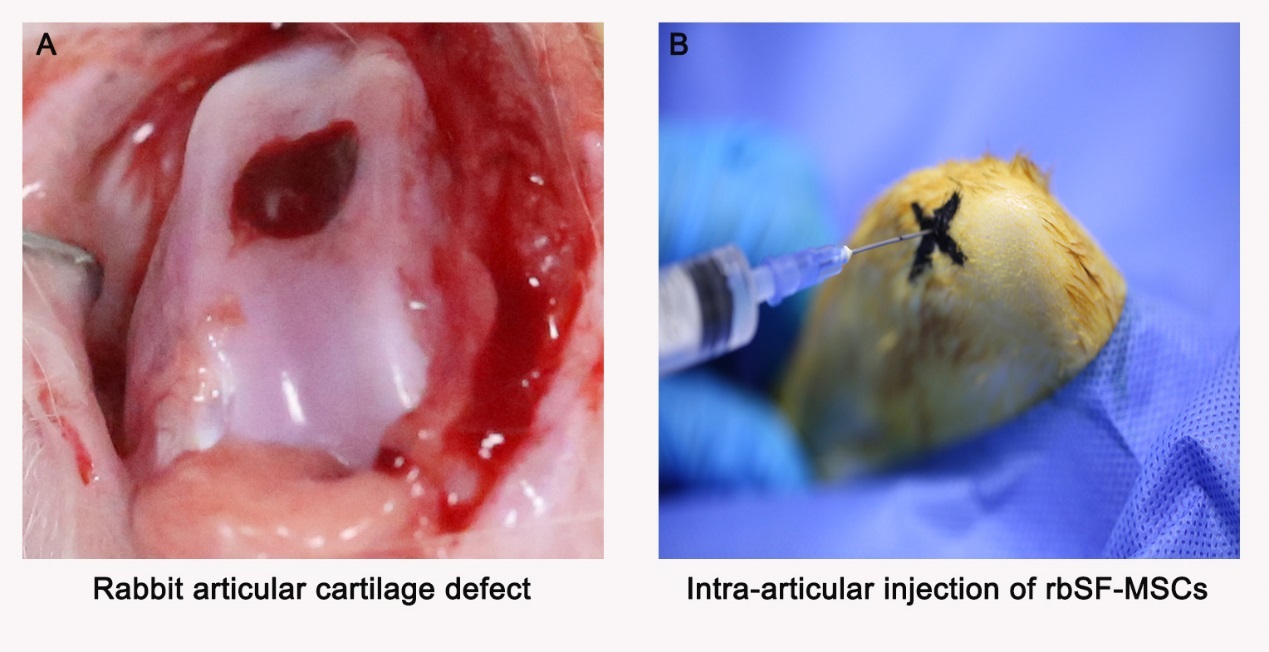

Supplement: Supplementary file 1 — Additional file 1: Figure S1. Surgical procedures of rabbit articular cartilage defect introduction and intra-articular injections of rbSF-MSCs. (A) A cylindrical full-thickness cartilage defect (3.5 mm in diameter and 1.5 mm in depth) was created on the trochlear groove using a special drill. (B) The cells were articularly injected in the knee joint of experimental rabbits using using large 18G size needles. [file 12967_2018_1485_MOESM1_ESM.docx]
