# Supplementary material for: Repair of articular cartilage defects with intra-articular injection of autologous rabbit synovial fluid-derived mesenchymal stem cells
Source: J Transl Med. 2018 May 9;16:123. doi: 10.1186/s12967-018-1485-8 (PMC5941664; doi:10.1186/s12967-018-1485-8)
Supplement: Supplementary file 2 — Additional file 2: Table S1. International Cartilage Repair Society macroscopic evaluation of cartilage repair. [file 12967_2018_1485_MOESM2_ESM.docx]

**Table S1. International Cartilage Repair Society macroscopic evaluation of cartilage repair**

| **Categories** |  | **Scores** |
| --- | --- | --- |
| **Degree of defect repair**  In level with surrounding cartilage  75% repair of defect depth  50% repair of defect depth  25% repair of defect depth  No repair of defect depth  **Integration to border zone**  Complete integration with surrounding cartilage  Demarcating border-1 mm  Three-quarters of graft integrated, one-quarter with a notable border-1 mm in width  One-half of graft integrated with surrounding cartilage, one-half with a notable border-1 mm  From no contact to one-quarter of graft integrated with surrounding cartilage  **Macroscopic appearance**  Intact smooth surface  Fibrillated surface  Small, scattered fissures or cracks  Several small or few large fissures  Total degeneration of grafted area  **Overall repair assessment**  Grade I: normal  Grade II: nearly normal  Grade III: abnormal  Grade IV: severely abnormal |  | 4  3  2  1  0  4  3  2  1  0  4  3  2  1  0  12  8-11  4-7  1-3 |
