# Supplementary material for: Repair of articular cartilage defects with intra-articular injection of autologous rabbit synovial fluid-derived mesenchymal stem cells
Source: J Transl Med. 2018 May 9;16:123. doi: 10.1186/s12967-018-1485-8 (PMC5941664; doi:10.1186/s12967-018-1485-8)
Supplement: Supplementary file 3 — Additional file 3: Table S2. The ICRS Visual Histological Assessment Scale. [file 12967_2018_1485_MOESM3_ESM.docx]

**Table S2. The ICRS Visual Histological Assessment Scale**

| **Features** |  | **Scores** |
| --- | --- | --- |
| **Surface**  Smooth/continuous  Discontinuities/irregularities  **Matrix**  Hyaline  Mixture: hyaline/fibrocartilage  Fibrocartilage  Fibrous tissue  **Cell distribution**  Columnar  Mixed/columnar-clusters  Clusters  Individual cells/disorganized  **Cell population viability**  Predominantly viable  Partially viable  <10% viable  **Subchondral bone**  Normal  Increased remodeling  Bone necrosis/granulation tissue  Detached/fracture/callus at base  **Cartilage mineralization (calcified cartilage)**  Normal  Abnormal/inappropriate location  **Toluidine blue stain**  Normal  Slight reduction  Moderate reduction  Severe reduction  No staining  **Percent toluidine blue in defect**  75–100%  50–75%  25–50%  0–25%  No toluidine blue staining |  | 3  0  3  2  1  0  3  2  1  0  2  1  0  3  2  1  0  2  0  4  3  2  1  0  4  3  2  1  0  Max 24 |
